# Researching Time and Ageism: Applications of Qualitative Longitudinal Research to the Field

**DOI:** 10.1177/07334648231160982

**Published:** 2023-03-03

**Authors:** Katri Keskinen, Pirjo Nikander

**Affiliations:** 1 Faculty of Social Sciences, 7840 Tampere University, Finland; 2 Doctoral School, 7840 Tampere University, Finland

**Keywords:** age norms, ageism, life-course, methodology, qualitative methods

## Abstract

Interest in ageism research has grown immensely since the term was coined. Despite methodological innovations to study ageism in different settings and the application of different methods and methodologies to the topic, qualitative longitudinal studies investigating ageism are still underrepresented in the field. Through qualitative longitudinal interview data with four individuals of the same age, this study explored the applications of qualitative longitudinal research on ageism, highlighting its potential benefits and challenges to the multidisciplinary study of ageism and to gerontological research. The paper presents four distinctively different narratives through which individuals “do,” “undo,” and “challenge” ageism in their interview dialogues over time. Doing this underlines the importance of understanding the heterogeneity and intersectionality among encounters, expressions, and dynamics of ageism. The paper concludes with a discussion of the potential contributions that qualitative longitudinal research makes to ageism research and policy.

**What this paper adds**

• Introduces readers to qualitative longitudinal research in the study of ageism.
• Undermines ageism by investigating heterogeneous life courses.
• Evaluates how qualitative longitudinal research informs future policies and practices addressing ageism.

**Applications of study findings**

• Challenges the use of arbitrary age limits in policy-making by showcasing the heterogeneity among individuals sharing similar life courses.
• Encourages further research on ageism through time to better understand the developments and processes through which ageism is constructed.
• Provides practical insights into how ageism can be studied through a qualitative longitudinal methodology.

## Introduction

Throughout the life-course, individuals engage in sets of age-appropriate behaviors shaping the normative life-course, reinforced by the formal and informal policies and institutions in place (Nikander, 2002). Not only do they define right time to do things, such as having children (Ylänne & Nikander, 2019), but they also teach us how deviating from age-appropriate behavior creates a moral obligation to explain oneself (Nikander, 2000). This paper approached ageism from a discursive perspective, seeing it as a social construct manifested deep within societies, and reproduced and reinforced by institutions that maintain ageist ideologies (Bytheway, 2005). Focusing on the dynamic interplay between individual identifications, organizational practices, and social structures allowed us to investigate how individuals “…do things with words, they can do ageism as well as undo and challenge it…” (Previtali et al., 2022. p. 11) in their everyday encounters and accounts. Ageism is often experienced when individuals step out of cultural, normative life-course timings and go against shared norms, expectations, and ideas of how and when to do things in life. Therefore, breaking “suitable” age codes and norms, such as when to have children (Ylänne & Nikander, 2019), engage in education (Leonard et al., 2018), or retire (Vickerstaff & Van der Horst, 2021) can all be seen as *wrongly timed actions, given your chronological age.*

By following the lives of ordinary people across time, we can better understand how individual experiences of aging are constructed to follow normative life-course and match age-appropriate guidelines. Qualitative longitudinal research (QLR) has the potential to track and closely examine the changes, transitions, and temporality in individual lives, as well as the meanings and interpretations individuals give to each of these changes. Recently, scoping reviews on ageism have underlined the need to research ageism longitudinally in different settings, such as in working life (Harris et al., 2018; Previtali et al., 2022), and during historical events, such as the COVID-19 pandemic (Werner & AboJabel, 2022). Despite the long-standing interest in qualitative longitudinal studies in gerontology, longitudinal studies investigating ageism have been predominantly quantitative in nature. However, the results from existing qualitative longitudinal studies have already shed light on the value of their contribution to the field. For example, a qualitative longitudinal study from Taiwan investigated the experiences of perceived ageism among older patients following hip fracture and found that over time, perceptions of experienced ageism changed from positive to negative (Huang et al., 2014). Although patronizing ageism was perceived as positive and caring during the first interview, these experiences were later characterized as turning points when individuals were deprived of their autonomy and power. Therefore, to create effective solutions that address ageism in different areas of life, researchers need to better understand the nature of ageism, especially in relation to time.

First, this paper begins with a discussion of the potential benefits and challenges of QLR on ageism. Second, it provides a practical example of how everyday experiences of ageism can be studied using QLR. Finally, it considers the contributions that qualitative longitudinal studies can make to the growing field of ageism research and policy.

## Benefits and Challenges of Qualitative Longitudinal Research

QLR, characterized as qualitative research involving two or more data generation time points over a period of time (Nevedal et al., 2019), QLR offers an opportunity to research not only the dynamics of individual lives (Neale, 2019), but also the various important areas of aging and gerontology (see Nevedal et al. (2019) for a review of qualitative longitudinal studies in gerontology). As the general benefits and challenges of QLR have already been discussed widely elsewhere (e.g., Hollstein, 2021; Neale, 2019; Thomson & Holland, 2003), our focus here is on the benefits and challenges of QLR on ageism research and policy.

The key complication for qualitative studies on ageism seems to be the lack of a unified definition of ageism, which, on the one hand, renders creating a unified theory more difficult, and on the other, offers freedom to methodological advances in the field (Previtali et al., 2022). Although QLR has received criticism for being obscure and too flexible in terms of its methodology, data sources used, and methods of analysis (Hollstein, 2021; Neale, 2019; Nevedal et al., 2019), its flexibility is particularly useful when investigating nuances of complex concepts such as ageism. At the core of QLR “lies a concern with the dynamics of human agency—the capacity to act, to interact, to make choices, and to influence the shape of one’s life and the lives of others” (Neale, 2019, p. 9). Therefore, it offers an excellent tool for investigating how individuals experience, challenge, undo, and do ageism in time and over time. Taking into account key events, turning points and accumulating experiences, QLR also offers a unique way to holistically understand the life-course and origins of ageism. In addition to individual accounts of ageism, qualitative longitudinal studies often collect various other forms of data, including newspaper articles, policy documents, observations, field notes and diaries that connect the researched timespan into its spatio-temporal context and allow researchers to investigate ageism and its origins in a more comprehensive manner.

Because our everyday experiences of age and ageism are constructed in their spatio-temporal contexts, researching ageism through time also presents challenges. It requires researchers to travel through time between past, present, and future interpretations, understanding ageism and experiences of it as both situationally constructed and shaped through time. Addressing change over time through QLR creates challenges, which is why ageism researchers have been reluctant to approach qualitative longitudinal datasets, for instance, from a discursive perspective (Previtali et al., 2022). In addition, QLR typically generates large amounts of data, which can prove overwhelming and lead to “death by data asphyxiation” (Pettigrew, 1995, p. 111), further complicating the research process of pinpointing ageism in the data.

Qualitative longitudinal studies also require resources, time, and commitment from funders, researchers, and the participants, which may be challenging to achieve. Although QLR has the potential to delve into the shaping causal processes and “what works?” in policy-making (Neale, 2021), qualitative longitudinal studies are more expensive than cross-sectional studies and do not always provide quick solutions. This means that the results of the research are available only after policymakers have decided on the continuance of the researched policy. However, investing resources into examining ageism in policy and practice through QLR offers a valuable opportunity to uniquely understand and address the hidden mechanisms and dynamics that produce and reproduce ageism over time.

## Data and Research Process

This paper explored the additional value that the QLR design can bring to the study of ageism. To achieve this, we utilized qualitative longitudinal interview data from a nationwide longitudinal project titled “Towards Two-Speed Finland?” This project investigated the lived experiences of individuals aged 50 years and over who experienced job losses after working for the same employer for a long time and examined their everyday lives, career choices, and agency after exiting long-term employment. In this article, we focused longitudinally on four (4) individuals of the same age (58 years) who lost their jobs after a long career, totaling at 20 interviews under analysis, generated throughout the research process. In QLR, studying a small number of cases can also involve large volumes in terms of data density and intensity, as the number of interview waves and different forms of data contribute to the insights achievable from the data (Thomson, 2007).

**Figure 1.**
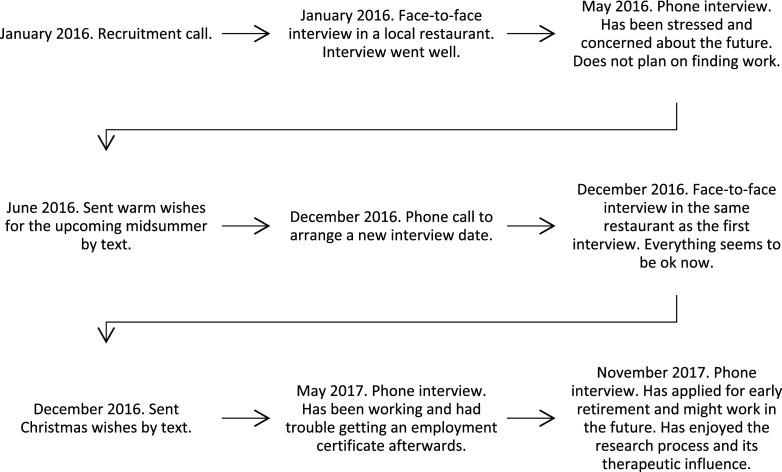
Illustrating the research process and timetable with one participant during qualitative longitudinal research.

The overall data was generated by two researchers who interviewed the participants between three and eleven times from 2015 to 2018 through face-to-face and phone interviews. A total of 183 interviews were conducted. The research process and its timetable are illustrated in Figure 1. Potential participants were contacted and recruited through name lists provided by their employer, the Finnish Postal Service. Additional participants were found through snowballing, as participants referred the researchers to their former colleagues and friends who were also interested in taking part, resulting in a total of 40 participants from different parts of the country. The research followed the ethical guidelines of the Finnish National Board on Research Integrity (TENK, 2019) and did not require an ethical review.

Each participant was first interviewed face-to-face in their home or in other places they preferred such as public libraries or cafés. Prior to each interview, the participants consented to recording the interviews, either in writing or verbally during the following interviews. The face-to-face interviews were semi-structured and attempted to capture the biographical work and life events that the participants considered important during their life-course. The participants were asked various questions, such as: *What has been the meaning of work in your life? How has your life changed now? How do you see your future?* To structure and facilitate memory work during the biographical interview, each participant was asked to draw two biographical maps of their adult lives, one each from their working life and private life, highlighting the life satisfaction they experienced at various life events. Introducing visual methods into qualitative interviewing not only facilitates memory work, but also allows illustrations of nonlinearity and multidimensionality during a life-course (Schubring et al., 2019), creating a more nuanced image of an individual’s life. Biographical maps, such as those used here, also work as interview props that can be referred to and used throughout the interviews.

Participants were then contacted through phone calls and emails during the follow-up period, depending on their availability and interest, resulting in one or more phone interviews. Toward the end of the research process, participants were invited to take part in final face-to-face interviews in their homes or preferred public spaces. During the final interviews, the interviewers reflected on the longitudinal interviewing processes with the participants and revisited and reflected on their first interviews. Using the previous biographical maps as guiding tools, the participants were asked to draw biographical maps of their financial situations during their adult life-course. All participants were given pseudonyms, and their interviews were audio-recorded and transcribed verbatim.

### Participants

Traditionally, postal service has been considered an organization that provides long careers and job stability. However, because of digitalization and a sharp decline in paper mail, there have been some organizational shifts during the past years, resulting in changing work demands and a high number of dismissals. Most of the participants had joined the postal service at a young age, gaining most of their education on the job during their working years. The four selected participants were all aged 58 years during their first interviews, and the lowest possible age at which they could receive an old-age pension was set to 63 years and 6 months. However, with a 6-month severance bonus from their previous employer, the participants were able to access an early exit through the unemployment pathway to retirement, a scheme established to secure income during unemployment near retirement (Kosonen et al., 2020). This meant that they were financially secured until they could apply for an old-age pension at a lowered age of 62 years. We selected the participants because despite sharing the same chronological age, they represented the diversity in the data regarding career choices and experiences following job loss. General information about the four participants is summarized in Table 1.

**Table 1.** General Information About the Participants During the Research Process. Age Was Reported as the Age at the First Interview.

Participant Pseudonyms	Vuokko, Woman	Kari, Man	Laura, Woman	Iida, Woman
Age during first interview	58	58	58	58
Previous work position	IT specialist	Delivery	Customer service	Customer service
Educational background	Comprehensive school	Post-secondary education	Post-secondary education	Comprehensive school
Family situation	Married, no children	Married, children, grandchildren	Married, children, grandchildren and parents needing care	Married, adult child, older parent needing care
Health status	No issues mentioned	No issues mentioned	No issues mentioned, less stress after giving up work	Mobility issues and long sick leaves preceding job loss
Length of career	38 years	38 years	40 years	40 years
Location	Major city	Major city	Rural town	Rural town
Work-life transitions during the data generation	From unemployment to part-time employment	Long-term unemployment with short contracts in between	Unplanned early exit	Planned early exit

## Methods and Analysis

The initial phases involved familiarizing with the data and participants by listening to the audio recordings of the interviews and reading through the interview transcripts many times. Together with the researchers’ field notes, the transcribed face-to-face and phone interviews were then summarized into researcher-constructed case profiles (Neale, 2019; Thomson & Holland, 2003) that chronologically captured both the participants’ future views and important life and working-life events. These tools provided a nuanced image of how the cases unfolded over time and facilitated case comparisons that enabled finding relevant snapshots of the data during later analysis (Neale, 2019, p. 112).

Following Thomson (2007), the case profiles were used as a starting point to construct more in-depth and focused case histories. Case histories are developed from descriptive case profiles and examine how actions and motives are framed, what the recurrent themes and phrases in talk are, and pinpoint the key events and turning points in the storytelling (Thomson, 2007). The interview accounts were then analyzed individually with a theoretical focus on normativity and ageism taking place in the emerging narratives. For each participant, this was combined with their key events, motifs, and framings creating longitudinal case histories. The constructed case histories are discussed in relation to ageism in the following sections.

## Findings

Through QLR, we investigated ageism among four people sharing the same age and job loss from the same employer. In the following sections, we provide case histories of the participants, with a focus on whether and how ageism is present in their narratives. To better understand the life-courses behind each interview account, we provide illustrations, and explanations of the biographical maps of work-life and overall life satisfaction during each participant’s adult years.

### Vuokko, 58

**Figure 2.**
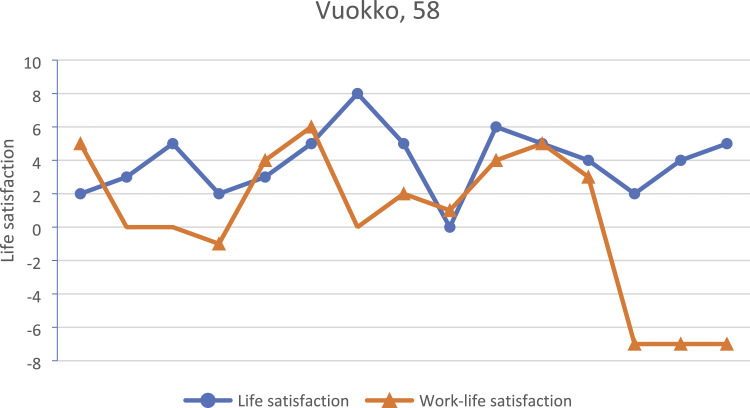
Vuokko’s biographical map of life and work-life events and life satisfaction in adulthood as drawn during the first interview.

Vuokko, 58, had worked in various positions during her 38 years with the postal service. During her adult life, she had experienced some downfalls, as portrayed in Figure 2, the biggest of which were not being able to have children of their own with her husband, and the death of her parents later in life. Not having children had made her dedicated to work, but the recent death of her parents made her undermine the importance of work in life. During her years at the postal service, she received in-house training and was able to advance between positions, which accounted for variability. However, because of the organizational shifts in the 2 years preceding her job loss, she had started to feel less and less satisfied at work. Her boss had changed, and her work demands had increased. In the end, her job contract was ended, and she was given 6 months’ pay.

I’m done with the postal service; now, it’s time for something new. But I don’t know what that something new is yet. Of course, I’ve applied for positions, but at least for now, I think it’s because I don’t know how to do it. I don’t know how to market myself because I’ve never needed to do that.

Vuokko had previously worked in information and communication technology, but having had only in-house training for the job, she lacked the formal education for positions that matched her experience. During her job search, she felt inferior, as most job openings required some level of higher education, something she lacked. Although she wanted to find a job, she had already started to consider replacing paid work with volunteering and other activities with her retired husband. A couple of months after the first interview, Vuokko heard from her friend about a job opening in a small company that was not publicly advertised. She applied for and got the job.

I feel great. Let’s just say, I finally found a company where age was not a problem [laughing]. Because it’s mostly been the issue—it always goes, “yeah, you’re too old, this is not going to work out.” They don’t use these words, but that’s essentially what it is.

Drawing from a commonly held understanding that older jobseekers are discriminated against in hiring practices, Vuokko ascribed her unsuccessful attempts to find a job to her age. In her previous interviews, she attributed her unsuccessful job applications to her lack of education and the know-how of applying and marketing herself as an employee. However, after a couple of months, her perception changed. Vuokko had originally hoped for a full-time job, but having talked with other jobseekers in her position, she had concluded that “*50+ women are poisonous*” in working life, which for her meant taking even the part-time job she was offered. Simultaneously, she looked back at her unemployment period as a positive experience during which she got to “*practice retirement*” with her husband. During her last interview, Vuokko shared her views on aging and the future.

At the same time, aging scares and doesn’t scare me. I’m disgusted that health worsens, but I hope to stay fit enough to cope. And, of course, by doing things for your own mobility, taking care of your health. Wearing glasses sucks. I don’t like these; these are the things I find boring about becoming old. I’m not worried about wrinkles—these kinds of things—but losing an ability. That’s not nice. But at the same time, I hope there will be more leisure time of your own. That’s what I’m waiting for. But, we’ve always been an active couple; we like to do and organize things even for others. It’s always us who organize. If not all of it, at least part of it [laughing]. I hope it continues the same way. I could easily learn a new language still because I like languages and find them easy, even now. Something like this, some kind of a hobby is a must.

Despite having a retired husband at home, it was clear to Vuokko from the beginning that she wanted to continue working longer. In her interview account, she drew on the belief that old age equals decline, vulnerability, and loss of functionality, fueled by her own recent experiences of losing her parents to illness. Although she did not mind looking older, as long as she could continue doing the things she enjoyed, Vuokko detested her own aging process and saw staying active as a means to avoid aging and the inevitable loss of functionality—something she found *disgusting* and *boring*.

### Kari, 58

**Figure 3.**
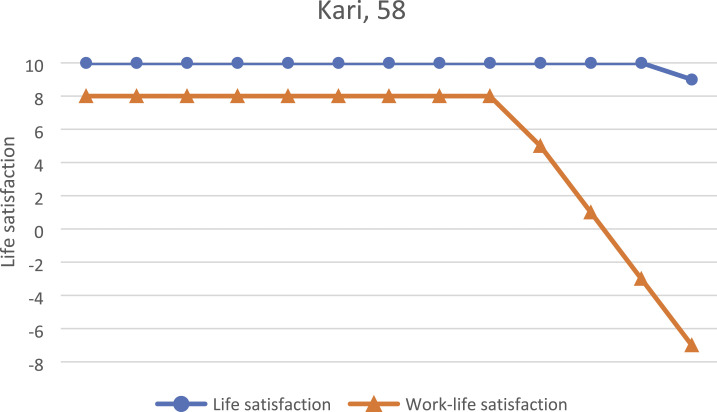
Kari’s biographical map of life and work-life events and life satisfaction in adulthood as drawn during the first interview.

Kari, 58, worked for the postal services for 38 years before he chose to leave his job during downsizing. Looking back at his life, he was content with the things he had accomplished, as shown in Figure 3. A couple of years prior to resigning, he had lost motivation to work for the postal service due to increasing work demands and enrolled in adult education while working. He and his wife owned a house near a large city and had children and grandchildren they wanted to financially support. Kari graduated shortly after his work contract was terminated and hoped to start working right after, but he soon found out there were no matching jobs available. However, he was hopeful about his re-employment after obtaining a new degree.

I had this idea then, with the career. When I was working for the postal service, the motivation was what **it** was, especially in the end. I thought that if I ever retire from that company, I won’t work a day more than I have to. But now, after the re-education, and with my wife being five years younger, I was thinking I could easily work another ten years, as long as health allows, so easily until 68.

Over the months, Kari started to feel that finding a permanent job was unlikely and thought about setting up his own company. He noticed that many companies hired independent contractors rather than permanently employed workers. Despite being an active jobseeker and constantly looking to improve his qualifications, Kari managed to find only short-term contracts. This meant having to constantly look for the next job opening and juggling between employment and unemployment.

… You could say that now, the possible, even the potential openings, jobs I’ve applied for, they’ve somehow disappeared. There haven’t been that many openings, but I guess it still seems to be a problem that, as an adult, or let’s say even at this age you start changing professions, without the work experience, what weighs in the most are age and lack of experience from the field. Little by little, I’m beginning to believe that the labor market here in Finland is no longer a place for people nearing their sixties.

Eventually, constant job searches and stress wore him down. Kari had previously hoped to work until the age of 68, but the idea started to seem distant over time. Like Vuokko, Kari characterized his age as a problem in his job search. Rather than challenging the idea that the labor market is no place for people nearing their sixties, Kari seemed to accept it as a fact. After encountering precarious and uncertain working conditions, Kari planned to retire as early as possible, not to withdraw from the labor market but to secure his finances for the rest of his working years and continue working on retirement.

I’ve been thinking, since I’m part of the last blessed age cohort, if I don’t work in between, I get extended unemployment benefits until I’m eligible for old-age pension. There’s a possibility I could retire at 62. I’ve been thinking about it. It’s a bit less than two years from now, and if things won’t change, I’ll retire. And the other thing is that if you’re on retirement, you can still work without having to worry about its effects on unemployment benefits. But once I’m retired, nothing will affect my pension. I could work a lot more, maybe even become an entrepreneur.

In the end, Kari felt that because of his age, the policies in place encouraged him to retire rather than continue working, undermining the commonly shared political goal of extending working lives. Instead of explaining his career decisions through ageism in the labor market, he perceived retiring as the most favorable option, enabled by the labor market policies that offered him a less problematic solution. In line with Vuokko, Kari’s perception of the future and retirement was filled with activities, in his case, working until his younger wife could also retire.

### Laura, 58

**Figure 4.**
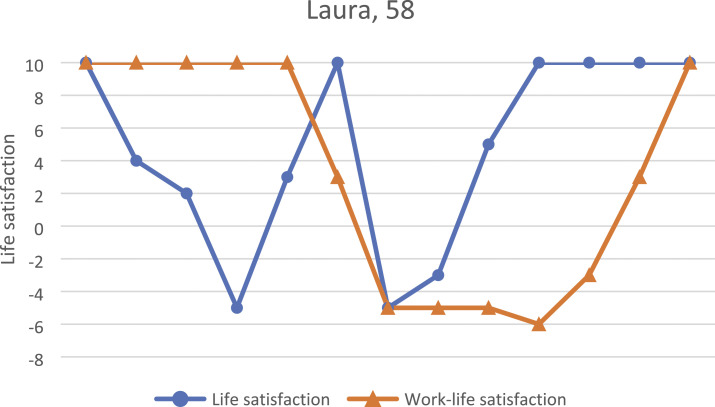
Laura’s biographical map of life and work-life events and life satisfaction in adulthood as drawn during the first interview.

Laura, 58, had worked at the postal service for 40 years before her contract was terminated due to downsizing. She and her husband owned a house together in a small city where she had worked all her adult years. Laura had received most of her education on the job and was able to change positions and develop inside the company during those years. Having older parents to care for and small grandchildren, she had been concerned about her own coping and considered applying for a part-time pension a couple of months before the company announced its downsizing decisions. Laura described her adult life as happy despite the stressful years when her children were small and when she was promoted to a supervisory role in her forties, as shown in Figure 4. The stress following her promotion influenced both her personal and working lives, and finally, she decided to request her previous position. Looking back at her previous stressful experiences, she attempted to keep her stress at a minimum.

The one thing I hoped from work was that I could cope with it all. And, of course, needing some kind of relief, somehow, I felt that I was just exhausted. And thinking at the same time, Mom, and Dad are still alive. I need to have energy to spend time with them. They’re both over 80 now. And then there’s the small grandchildren, having the energy to be with them and do stuff.

Having her everyday life filled with caring for her family members, Laura was happy that her income was secured until retirement through her early exit. As the months passed, she had noticed she slept and felt better than in years, and in some way, the job loss had been a blessing in disguise, as it allowed her to spend more time with her family. She was interested in learning a new language and joining different volunteering activities, but as time passed, her parents needed more and more help.

Now that things are possible, when you’re no longer in working life, I’m really interested in learning something new or doing something, or joining an activity, but I have to say now it has been so busy. As I said, Mom, and Dad, and now with grandchildren and everything so, I haven’t even had the time to think about what I want to do.

At the end of the data generation period, Laura had applied for an early part-time pension, and her everyday was filled with informal care responsibilities. She no longer felt sad about losing her job even though she missed the interactions she had with customers. From the beginning, it was clear to Laura that she would not want to apply for jobs. She had worked a couple of days during the elections, which had resulted in a large amount of paperwork and clarifications—something she wished to avoid in the future. The experience made her realize that getting back to working life would be more problematic than retiring, similar to Kari’s experience.

### Iida, 58

**Figure 5.**
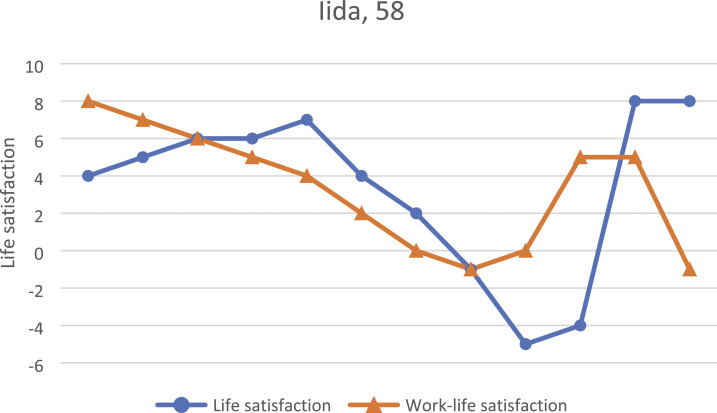
Iida’s biographical map of life and work-life events and life satisfaction in adulthood as drawn during the first interview.

Iida, 58, had worked for nearly 40 years at the postal service when her workplace was closed due to downsizing. She described her life as happy; having children and meeting her current partner were especially joyous moments in her life, as illustrated in Figure 5. She had always enjoyed work until she started having health problems that had caused problems for both her work and her personal lives. Preceding her job loss, she had taken many lengthy sick leaves due to health problems. Fortunately, she was just the right age to access early exit because soon after she lost her job, her mother’s health began to worsen, taking a sizable portion of her time.

… Time is flying. I haven’t missed work, and the biggest reason for that right now is mostly that our mom, she’s been in quite a bad shape most of the spring and winter, so basically during the days, I’ve been almost like a full-time caregiver. Siblings take care of the evenings, nights, and weekends, but I’ve had things to do.

Eight months later, Iida and her siblings found an assisted-living facility for their mother, and she finally had time to think about the things she wanted to do now that she was out of work. She and her recently retired husband had long planned on a holiday in their cabin, and they looked forward to taking the time to rest.

Now that I think about the upcoming summer, already last summer I thought we get to spend it at our cabin, not needing to leave the place more than once a week for groceries, so now, in principle it might even work now that Mom is in good care, I have time to myself with no rush. It’s the, I don’t need any travels or extreme [laughing]. I’m just happy when I get to rest and knit socks.

A year after the first interview, Iida reflected on her career at the postal service, a place she had considered her second home for 40 years. For her, it was clear from the beginning that she would not look for another job, as her work identity had been built around being a postal worker. As a sign of ending her career, she had planned to burn her work uniform and start a new chapter in her life.

… I know that I have full right to live on unemployment benefits, I don’t have a guilty conscience or feel that I should, that I’d be obliged to do something else. I’ve just thought, I have unemployment security; I am the fitting age and been working enough years. In my case, I don’t feel bad about not being an active jobseeker. I’ve internalized that feeling of freedom now. I can leave as I want, do what I want, of course, within the financial constraints that allow, but it doesn’t oblige me to leave home at a certain time. The only things with schedules now are dentist and optometrist appointments; the rest I can do as I please.

Similar to Laura’s situation, Iida also replaced paid work with informal care. After finding her mother an assisted-living facility, Iida could no longer explain her work exit, drawing on the belief that women should take on informal care of their older parents. Instead, she drew on her long working career and age, which enabled her early exit from working life. However, acting against the political goals of extending her career until she attained the age of 63 years and 6 months still pressured Iida to defend and explain herself, as if she was wrong for choosing to retire at her age.

## Discussion

We employed a qualitative longitudinal methodology to research ageism in the interview accounts of four individuals who shared the same chronological age and former employer. Despite sharing these characteristics, the life stories and decisions of each individual following job loss differed greatly, undermining the use of homogeneous concepts and arbitrary age limits in policy-making that time life events and transitions. Individuals drew on ageism to explain their unsuccessful job search, their need to stay active, and their decision to retire earlier than planned. Even though the participants fitted the age to exit early, there was a need to explain deviations from the political goal, whether it was through continued activity on retirement, informal care, or through a long career. Simultaneously, old age was detested and perceived as a decline in health and activities, something the participants wished to avoid in the future.

This paper has only scratched the surface of what QLR has to offer in the field of ageism. Despite the existing challenges, QLR provides novel and promising perspectives for the rapidly expanding field of ageism research. First, QLR has the potential to answer questions such as when, why, where, and how ageism unfolds and is experienced through time and life-course, and as such provides a comprehensive and versatile methodology able to uncover ageism in everyday life, policies, and practices. Second, QLR contributes to the understanding of ageism and life-course by underlining how life events and accumulated experiences contribute to the internalization and perceptions of ageism. Third, QLR provides tools for political efforts and policy programs concerning age and for addressing short- and long-term effects and potential ageism.

Given the pervasiveness of ageism and age inequality, qualitative longitudinal studies yield crucial insight into the dynamic interplay between individual pathways and decisions, organizational practices, and social structures, and how ageism operates within and between them. Furthermore, understanding key life events and cumulative effects taking place during the life-course is crucial to understanding why individuals engage in ageist behavior and exclude themselves from certain activities in later life. Therefore, more research that engages with ageism using QLR designs is urgently required.
